# Deep learning: a new tool for photonic nanostructure design

**DOI:** 10.1039/c9na00656g

**Published:** 2020-02-12

**Authors:** Ravi S. Hegde

**Affiliations:** AB 6/212, Indian Institute of Technology Gandhinagar Gujarat 382355 India hegder@iitgn.ac.in +91 79 2395 2486

## Abstract

Early results have shown the potential of Deep Learning (DL) to disrupt the fields of optical inverse-design, particularly, the inverse design of nanostructures. In the last three years, the complexity of the optical nanostructure being designed and the sophistication of the employed DL methodology have steadily increased. This topical review comprehensively surveys DL based design examples from the nanophotonics literature. Notwithstanding the early success of this approach, its limitations, range of validity and its place among established design techniques remain to be assessed. The review also provides a perspective on the limitations of this approach and emerging research directions. It is hoped that this topical review may help readers to identify unaddressed problems, to choose an initial setup for a specific problem, and, to identify means to improve the performance of existing DL based workflows.

## Abstract 2

We review recent progress in the application of Deep Learning (DL) techniques for photonic nanostructure design and provide a perspective on current limitations and fruitful directions for further development.

## Graphical abstract

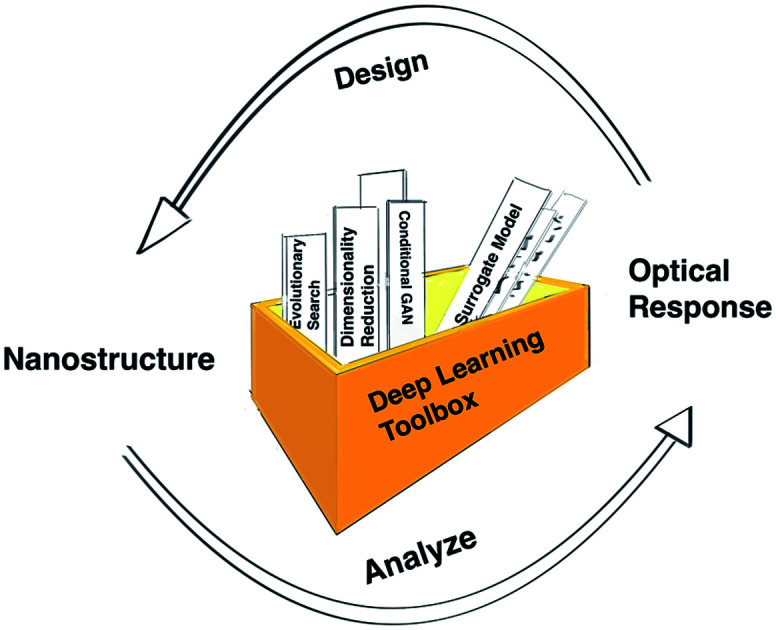

## 1 Introduction

The last decade has witnessed a revolutionary development in the form of Deep Learning (DL),^1,2^ a data-driven technique that uses a hierarchical composition of simple nonlinear modules. The broad popularity of data-driven techniques like DL has led to the development of Scientific Machine Learning (SciML),^3^ a field that aims to refine and apply data-driven techniques to tackle challenging problems in science and engineering.^4^ Noteworthy uses of data-driven tools include the identification of energy materials^5–8^ by accelerating searches^9^ and the prediction of the results of quantum simulations.^10^

Nanophotonics research^11^ is becoming more computation intensive.^12,13^ State-of-the-art nanofabrication technology allows unprecedented lateral resolution and stitching accuracy for wide-area patterning and the ability to stack aligned nanopatterned layers. The large number of spatial degrees-of-freedom is complemented by the wide choice of materials: plasmonic metals, high-index semiconductors and exotic two-dimensional materials to name a few. How do we explore this vast combined space of materials and structures efficiently? It is clear that novel computational techniques are needed for this task to become tractable. In addition, techniques^14^ are needed to assess which of the possible material/structure designs discovered computationally are likely to be experimentally realizable.

Formal solution techniques for the inverse problem of structural/material design are thus becoming increasingly relevant.^12,13^ The review by Campbell and co-workers^13^ provides a detailed account of the broad range of formal methods relevant to nanophotonics. Despite advances in computational power and the availability of a wide variety of such formal methods, inverse problems (especially those involving large degrees of freedom) remain challenging in many cases and even intractable in some cases. This is due to the exponential explosion of the search space volume with a linear increase in dimensionality (the so-called “curse of dimensionality”^15^) and the non-convex nature of most nanophotonics optimization problems. Optimal photonics design is thus either restricted to limited searches in global space (limited due to the large number of computations required) or to gradient based local searches that tend to get stuck at local optima. In this context, the developments in data-driven techniques like DL are attractive as they could potentially aid nanophotonics design by complementing (or, in some cases, supplementing) existing optimization techniques.

### 1.1 Aims, scope and organization

The current burst in activity and promising early results from photonics researchers indicate the upcoming role of data-driven techniques alongside theory and numerical computing. Three reviews^13,16,17^ closely related to this topic are found in the literature. Yao and co-workers^16^ summarized recent advances in the emerging field where nanophotonics and machine learning blend. A single section in this review was focused on optical nanostructure design and it managed to cover a few early papers only. Campbell and co-workers^13^ presented both an introduction to and a review of several of the most popular techniques currently used for meta-device design. The application of DL to nanostructure design received only a passing coverage in this review. The perspective article by Zhou and co-workers^17^ broadly looked at the emerging role of data-driven techniques focusing more on the discovery of new optical materials rather than optical nanostructure design. The fast-moving nature of this area has led to a rapid surge in the number of papers, increasing sophistication of the DL methodology and application to newer design problems. The motivation for this minireview is that a comprehensive survey of published nanostructure design examples and DL methodological variations would benefit new and existing researchers to identify gaps in the literature and to better direct their research efforts.

The first aim of this minireview is to comprehensively survey design examples and DL methodological variations that have appeared in recent literature. Due to the large number of papers under consideration, it is important to categorize them appropriately to derive insights. The first way to categorize the surveyed papers is to group them on the basis of DL methodology irrespective of the optical nanostructures considered. An alternative way is to group them based on the optical nanostructure being designed irrespective of the DL methodology employed. Both these classification schemes have their advantages and disadvantages.

The first classification is motivated by the fact that similar DL methodologies have been applied with minor variations to different optical problems. Geometry encoding, network architecture, and inversion schemes are some aspects that can be used to differentiate DL methodologies. The advantage of this classification is that it is clear-cut. Unfortunately, quantitative metrics like DNN training error, test error and such technicalities may not lend themselves to easy comparison across different papers. In other problem domains (like computer vision), researchers compete on public challenges (same problems) and standard public datasets allowing an easy assessment of the relative contributions of a particular paper. Such common problems and datasets have not evolved in the optical nanostructure community and neither is software and data sharing universally practised. The second classification scheme, if possible, wound bring the optical problem to the fore and permit comparison of the cost/benefit trade-off of various DL methodologies. Although, this classification is less precise than the first, it is not entirely arbitrary. We will argue later that structure–response mappings exhibit similarities that can be exploited for this classification. The approach adopted in this paper is to first look at the various DL methodological variations encountered in the nanophotonics literature in Section 2. Subsequently, in Section 3, the emphasis shifts to optical nanostructure design where four categories of nanostructures are considered.

Whereas the focus of the early papers was on demonstrating the utility of this technique, the concern now should be to establish the limitations and range of validity of these techniques^18^ and an understanding of the advantages and disadvantages in relation to existing approaches. Other problem domains have seen the application of DL techniques for a longer time period compared to the domain of optical nanostructure design. These resources provide a perspective on the challenges and promising research directions (see Section 4). Finally, in the conclusion section of the paper (Section 5), we identify some unaddressed problems and speculate on upcoming developments.

This minireview article is primarily intended for researchers who use computational techniques to design and optimize geometries for nanophotonics and integrated photonics for applications in sensing, energy harvesting, imaging and spectroscopy. Metamaterials and metasurface design concepts are also of interest to RF and microwave engineering communities as well as acoustic metasurface researchers. The minireview assumes readers' familiarity with the DL basics, terminology and software tools. For gaining familiarity, we note that there are already multiple resources devoted to DL techniques^2^ as well as a few which consider the application to problems in other science and engineering disciplines.

## 2 Role of deep learning

**Fig. 1:**
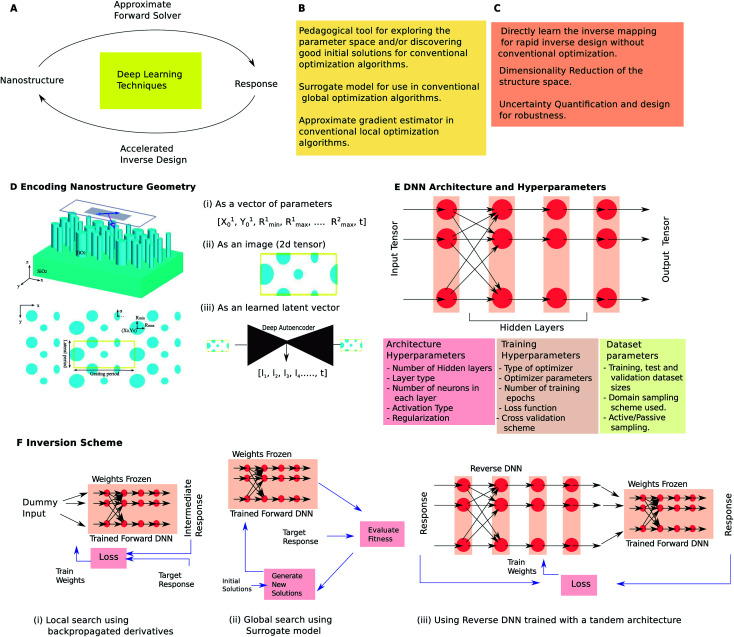
Overview of the role of deep learning in optical nanostructure design and summary of methodological variations used in nanophotonics design. (A) DL techniques can be used to obtain an approximate forward mapping (obtain optical response given a nanostructure specification) or *vice versa*. A list of some conventional (B) and unconventional (C) design tasks for which DL has been applied in nanophotonics design. (D–F) pictorially depict some of the methodological variations encountered in the encoding scheme, network architecture and inversion scheme. The inversion schemes shown in (F) use a fully-trained forward DNN with its weights frozen. See text for detailed description.

The relationship between a structure and its electromagnetic response (the forward mapping) is determined by the well-known Maxwell's equations which are accurate but computationally expensive to calculate in all but the simplest of geometries. The inverse problem, *i.e.* determining a nanostructure whose response closely matches a targeted optical response (the reverse mapping), is even more computationally expensive as it requires several point evaluations. Deep learning techniques are generally used in problems where the mapping between the input and output is unknown/impossible to estimate. The motivation of using DL is that approximate mappings can be “learned” (see Fig. 1A) and can be used to accelerate optical nanostructure design tasks considering that even large DL models can run with remarkable efficiency.

Fig. 1A considers the supervised learning paradigm where a set of input–output pairs is used to train a Deep Neural Network (DNN). Forward DNNs learn to predict the response given the structure (reverse DNNs do the opposite). The reverse mapping is generally one-to-many unlike the forward mapping which is always one-to-one making it difficult to directly train reverse DNNs. Forward DNNs can be used as a surrogate model in conventional local and global optimization workflows (Fig. 1B). Additionally, DL can enable novel and unconventional possibilities (Fig. 1C). If a reverse DNN can be trained somehow, it will completely obviate the need for optimization and can solve inverse problems within seconds. Many of the unconventional applications rely on advanced DL concepts like unsupervised learning, generative networks and network of networks.

The first step in applying DL to a design problem is to encode the structure and response into appropriate tensors. Three commonly encountered ways to encode the geometry are seen in Fig. 1D. The simplest technique is to parametrize the geometry and collect these parameters in a 1D tensor. While this is the earliest and most commonly encountered encoding scheme,^19–22^ its main drawback is that it drastically restricts the set of possible designs. For instance, Fig. 1D(i) restricts the set to a unit cell with a fixed number of elliptically shaped nanorods. Since it is not known whether a given response is even achievable with a particular shape, this encoding could lead to wasted efforts. On the other hand, an image like representation^23^ (2D tensor) can be used as seen in Fig. 1D(ii). The top view is pixellated and each pixel value represents the material present at that location.^24^ This representation preserves local information and is a nearly exhaustive (depending on the pixel density) set. However, the disadvantage is that many members of this set are completely unfeasible geometries. Large training sets and very long duration training are needed to ensure acceptable accuracy. A third alternative is opened up by using unsupervised learning with a deep autoencoder^25–28^ (Fig. 1D(iii)). Using the autoencoder it is possible to restrict the set of geometries to those which are suitable. The encoder part of the trained autoencoder is used to generate a latent vector to represent a shape. The encoding for the response space can be similarly chosen. Spectra and field distributions are the most commonly encountered responses. In structures where the response is dependent on incidence conditions (incidence angle, polarization, *etc.*) tensor representations (using the channel index) can be used.

After a suitable encoding is chosen, a suitable network architecture is defined; a dataset is generated; the dataset is split into train, test and validation sets; and, training and validation are carried out until acceptable error levels are reached. The trainable parameters should be distinguished from the so-called hyperparameters many of which are shown in Fig. 1E. A simplified view of a DNN architecture is seen in Fig. 1E which is a nonlinear function that maps an input tensor to an output tensor. The nonlinear function is compositional in nature and can be thought of as a sequence of layers. Feedforward DNNs are a particular class where data flow sequentially from left to right; in general, non-sequential data flows are also possible. The neuron is a key element of the layer which performs a weighted sum of some or all of the outputs of the previous layer and applies a nonlinear activation (modern DNN architectures allow neurons to accumulate output from neurons in multiple layers).

A fully connected layer has neurons which take input from outputs of all the neurons in the preceding layer. A DNN consisting of fully connected layers is a commonly used architecture^21^ and is especially suited when the geometry encoding is a vector of parameters. A convolutional layer has neurons which share the weights with all other neurons in that layer and which take inputs from only a selected set of neurons. DNNs containing convolutional layers are usually called Convolutional Neural Networks (CNNs) although these usually also contain some fully connected layers at the end. CNNs are well suited for problems where image-like encodings are used.^23^ Networks containing other types of layers like residual layers^29^ and those which are classified as Recurrent Neural Networks (RNNs)^23^ have been infrequently used in optical design. The choice of hyperparameters is itself a challenging optimization problem requiring multiple iterations of the define, train and test steps. A grid search with cross validation is the typically employed method to arrive at a suitable set of hyperparameters. The choice of hyperparameters influences the testing accuracy of a trained DNN; Hegde^29–31^ considered a problem (the design of thin-film multilayered antireflection coatings under normal incidence) to examine the effect of hyperparameter choice on testing the performance of a forward DNN. While larger models with large datasets can certainly improve testing accuracy, this has to be balanced against the cost of dataset generation and hyperparameter optimization.

In most applications, inverse design is the sought after goal. Fig. 1F shows three commonly encountered inversion schemes. Using the forward DNN as a surrogate is the simplest inversion approach due to the difficulty encountered in training a reverse DNN. Local optimizations require a gradient calculation to navigate the fitness landscape. Note that training of a DNN is also a local optimization which uses numerically determined gradients calculated using the backpropagation algorithm. A clever trick^21^ uses an already-trained forward DNN and creates a new DNN by adding a dummy input layer (with a single input of 1) at the input (Fig. 1F(i)). All the weights except the weights connecting the dummy inputs are frozen. Any set of weights thus represent a geometry and training the new DNN is akin to a local search in the structure space. The output of this network can be compared against a target response to provide a loss function against which the weights can be trained. Alternatively, the surrogate DNN can be used for the fitness evaluation step in a conventional global optimization routine^30,31^ as shown in Fig. 1F(ii). The saving in computation must be considered in light of the cost of training-set generation which will be amortized over several repeated optimization runs. Even in cases where such multiple runs are not needed, it should be noted that the training dataset generation is embarrassingly parallel as opposed to a typical optimization run which is sequential.^21^ The automatic numerical differentiation with respect to inputs is especially advantageous when compared with adjoint methods which require handcrafted objectives.

**Fig. 2:**
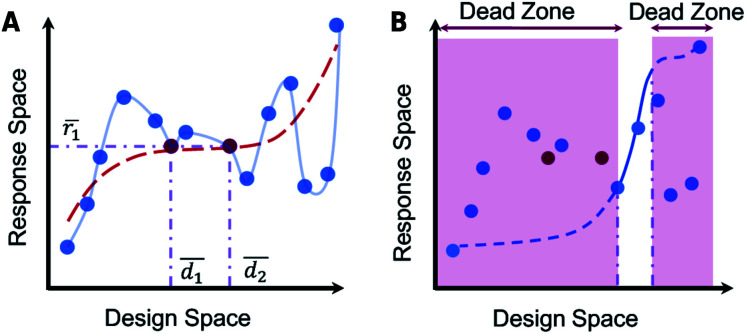
The problem of non-uniqueness. (A) A schematic representation of a general one-to-many design manifold (red dots are instances where two designs give the same response). A forced one-to-one mapping imposed on this manifold is represented by the red dotted line. (B) The creation of dead-zones due to the imposition of a forced one-to-one mapping is illustrated. Reproduced with permission from ref. 32 under the Creative Commons license.

The difficulties encountered in training a reverse DNN arise from the many-to-one nature of the reverse mapping and the fact that neural networks are by nature one-to-one mapping functions. Fig. 2 illustrates the non-uniqueness problem pictorially. A given structure has a unique optical response, but several structures may provide nearly similar optical responses. Some papers have reported the direct training of reverse DNNs without using any special techniques;^22^ this is possible if the reverse mapping is one-to-one to a large degree. The problem will be most noticeable when the training data contain samples where the designs are starkly different and the responses are nearly identical, leading to a convergence failure during training.^33^ In some problems, pruning the training dataset to not include such instances can allow the reverse DNN training to converge (*i.e.* dropping some samples).

The tandem network approach reported by Liu and co-workers^33^ is an improved method to train reverse DNNs. The tandem-network is a new DNN obtained by coupling an untrained reverse DNN and a fully-trained forward DNN (with frozen weights) as seen in Fig. 1F(iii). The tandem-network optimizes over a loss function which is smoother compared to training a reverse DNN directly. After training, the reverse DNN can be detached from the tandem-network and used on its own to provide rapid inversion. However, sample-dropping and even the “tandem-network training”^33^ approaches end up forcing a one-to-one mapping (Fig. 2A) which results in design “dead-zones” where the optimal design is unreachable (Fig. 2B).

Yet another way to train a reverse DNN is to use adversarial training.^25^ This approach differs from the tandem training approach in two ways: (1) the reverse DNN (called a “generator”) takes a latent vector in addition to the response tensor as input, (2) the training loss involves an additional term that aims to push the generator towards outputting feasible geometries. The use of the latent vector enables us to obtain a many-to-one mapping (different latent vectors combined with the same response function can give different structures as outputs). The dataless training methodology^34^ is a further variant of using generative networks for inversion.

It is seen that inversion techniques can be broadly classified depending on whether they involve the training of a reverse DNN or not. The techniques involving the reverse DNN have the clear advantage in inversion speed but impose a large development burden. Specifically, they are often limited by the accuracy of the trained forward DNN on which they depend for the training. Hu and co-workers reported that adding random noise during training improves the robustness of the obtained reverse DNN.^35^

## 3 Survey of designs

In this section, the surveyed papers are classified into categories based on the optical nanostructure considered for design with details of the DL methodology and comparative analysis. To explain the intuition behind the grouping, consider the example of the first category, isolated nanoparticles and core–shell nanoparticles. The optical response of these nanostructures is characterized by the presence of a few well-defined peaks. The structure can also be defined in terms of small one-dimensional vectors. Thus from the point of view of machine learning, this implies that a model with a relatively smaller representational capacity may be suitable. Indeed, papers have consistently reported excellent training and generalization errors for such problems. Consider, in contrast, the problem of multilayer thin-film design. Although, the structure can still be defined as a low one-dimensional vector, the spectral response is much richer. It is expected that inversion for this class of structures will be harder.

### 3.1 Isolated nanoparticles

Plasmonic,^37^ all-dielectric and quantum-dot nanoparticles and their collections are an important subclass of optical nanostructures. The optical response of isolated nanoparticles is relatively easy to compute. The optical response of these shapes exhibits a rich variety including ultra-high field enhancement^38^ and directional scattering.^39^ This problem is thus an ideal starting point for investigating the utility of DL techniques. The input geometry is easily encoded in the form of a small 1D vector (dimensions ≤ 16). The responses of interest are the far-field spectra and also the field distributions in the immediate vicinity of the nanoparticle at the resonance wavelengths (from which other quantities of interest like hot-spot strength can be assessed). We note that the spectra typically contain a small number of well-defined peaks whose center-wavelengths are strongly related to the geometrical parameters.

**Fig. 3:**
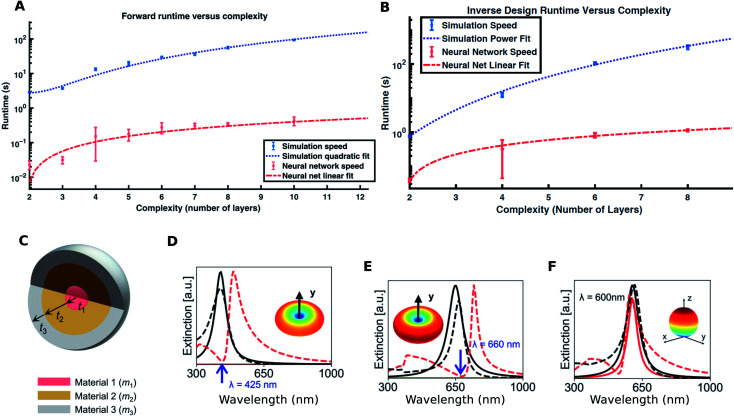
Application of DL to nanoparticle design: (A) forward DNN runtime for the same prediction error and (B) inverse design runtime improvements using the forward DNN as a function of geometric complexity of the structure. Reproduced with permission from ref. 21, ©2018, American Academy for Advancement of Science under the terms of the Creative Commons Attribution-NonCommercial license. (C) Geometry of a 3-layered core–shell nanoparticle with changeable materials. (D, E, and F) show the electric and magnetic dipole resonances of an inverse-designed particle (dashed lines) compared with the desired target spectra (solid lines). Insets show the 3D radiation pattern of the designed nanoparticle at the resonance wavelength. Reproduced with permission from ref. 36, ©2019, American Chemical Society.

The simplest of the shapes is a spherically symmetrical multilayered nanoparticle (“core–shell”). In their seminal paper, Peurifoy and co-workers^21^ considered a silica–titania multilayered particle with up to 16 layers to demonstrate the possibilities offered by DL. A feedforward DNN with fully connected layers was first trained to learn the forward mapping; inversion was achieved by using the scheme shown in Fig. 1F(i). The authors trained forward DNNs for particles with different numbers of layers. Trial and error was used to determine the optimal number of neurons in the hidden layers (number of hidden layers was fixed). The representational capacity required to “learn” the forward mapping is seen to increase with the number of layers. The fact that relative error can be minimized well below 1.0% with a small number of training samples (≈200 000) with moderate network sizes indicates that this mapping is easily learnable. This is also corroborated by the generalization ability of the DNN demonstrated by the authors. The scaling of the forward DNN runtime (for the same prediction error) and the inversion runtime seen in Fig. 3A and B respectively shows nearly two orders of magnitude speedup.

A more practical problem is to train a network to predict the response of practical core–shell nanoparticles (with up to 3 layers) for a wide range of material combinations. In their work, So and co-workers^36^ considered 3-layered core–shell nanoparticles where each layer can be one of 6 possible materials (Fig. 3C). Considering that plasmonic and high-index dielectrics were used, this covers a wide range of optical responses. The geometry encoding uses a combination of real numbers and factor variables (where the real numbers are the sizes and the factor variables denote the material used for the layers). The network was a feedforward DNN with fully connected layers and the inversion scheme used a tandem-network trained reverse DNN. A hand-crafted loss function was used to train the tandem network considering the mixed real number/factor encoding of the geometry. Training dataset sizes of ≈20 000 were used to train the network for ≈20 000 training epochs. The test MSE (mean squared error) of about 0.009 shows that adequate “learning” was achieved (a detailed discussion of the influence of training dataset sizes on test errors is found in ref. 31). The trained reverse DNN can be used to rapidly search for designs which match a targeted spectral response. Fig. 3D–F show the use of this tool to search for a core–shell nanoparticle whose electric and magnetic dipole resonance wavelengths can be independently designed. So *et al.* reported that some target spectra could not be achieved by this tool and speculated that this could be due to the fact that such a design does not exist for the parameter ranges chosen by them; however, they did not compare this inversion with a traditional optimization tool.

The prediction of field enhancement at the near-field hot-spots is important for the design of plasmonic sensors. He and co-workers^40^ show that DNNs can be trained to predict the electric field distributions in the vicinity of nanoparticles excited at the resonance wavelengths. They have considered spherical nanoparticles, nanorods and dimers of gold for this study which are simple shapes. The notable feature of this work was that the authors were able to significantly reduce the amount of training data needed *via* screening and resampling methods. It remains unclear whether such a procedure can be extended to complicated shapes or to particles with multiple materials as the dataset generation requires human involvement.

### 3.2 Multilayered thin-films

The design of multilayered thin-films, in particular, the problem of broadband antireflection coating (ARC) design has received extensive attention from researchers^41–45^ and a broad range of theoretical and computational techniques^45–49^ have been applied to it. Many high-performance commercial tools are available to design multilayered structures. From a DL point of view, we note that this is a challenging non-convex multi-modal optimization problem with regions of flat fitness.^41,45,50^ Strong mathematical and computational evidence points to the existence of global optima.^42–44^ Although this problem is superficially similar to that discussed in the previous subsection, it is noted that the spectral response can vary widely in comparison. This is especially true when the range of layer thicknesses is made larger and when high index materials are used. Additionally, highly different geometries can give very nearly the same spectra^33^ and make the inversion difficult.

Liu and co-workers^33^ considered a dielectric multilayer geometry and used a tandem-network based training to obtain a reverse DNN that can perform inversion rapidly. In the case of a graphene–silicon nitride multilayer geometry, Chen and co-workers^51^ considered the direct training of a reverse DNN using adaptive batch normalization (BN) techniques. Their results show that the network using adaptive BN outperformed the other alternatives. The possible explanation is that adaptive BN reduces the overfitting problem although it is not clear why regular batch normalization performed worse. These two papers have not compared the efficacy of the reverse DNN with conventional thin-film design tools.

**Fig. 4:**
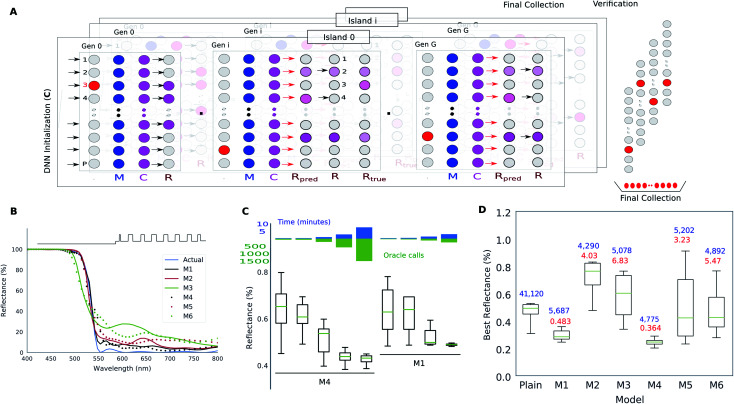
Thin film multilayer design using the DNN-surrogate assisted evolutionary search. (A) shows the overview of a multiple-island Differential Evolutionary (DE) algorithm where the repopulation phase (*R*) utilizes a DNN surrogate. (B) Comparison of the predictive power of six different DNNs which vary in the model complexity, training dataset size and training dataset bias. (C) Optimality, runtime and exact function call statistics for the evolutionary search run entirely on the DNN surrogate for two different DNNs. (D) Optimality and function call statistics for the DE run using DNN surrogates only for candidate preselection. Reproduced with permission from ref. 31, ©2018, IEEE.

Hegde^30,31^ adopted an approach to the inversion using only a trained forward DNN paired with the evolutionary search. The schematic of this approach is detailed in Fig. 4A which is a typical Differential Evolution (DE)^52^ optimization run. During each iteration of the DE, a repopulation phase requires that the child population is compared with the parent population which involves the estimation of the fitness of each child. This fitness estimation can be done in three alternative ways: (1) exactly using a so-called “Oracle”, (2) approximately, using a forward DNN, and (3) exactly using the oracle but only on a reduced set preselected by the DNN. Hegde^30,31^ evaluated the optimality and runtime metrics of the optimization for each of the three alternatives. Furthermore, they also considered how the hyperparameters of the forward DNN influence the optimization outcome. They trained six different forward DNNs which vary in aspects like training dataset size, model complexity and dataset selection bias. Fig. 4B shows that models trained on bigger datasets perform better, but, interestingly, the “worse” DNNs also tend to approximate the correct spectrum. Fig. 4C shows that the approximate fitness landscape of forward DNNs diverges significantly enough that an exhaustive search does not yield optima close to theoretical bounds (which are about 0.1% reflectance for this material system). Fig. 4D shows the surprising result that even “worse” DNNs can accelerate the evolutionary search when used in the preselection mode. In a different paper, Hegde^30^ compared the performance of a DL based design method with an open-source implantation of the needle-point method.

### 3.3 Periodic metasurfaces

Metasurfaces, two-dimensional arrays composed of subwavelength sized meta-atoms, manipulate light by imparting local and space-variant changes on an incident wavefront.^53,54^ Nearly, all properties of electromagnetic waves like amplitude, phase, polarization, spectrum, *etc.* can be manipulated by the metasurface. This has motivated the design of metasurface based devices like metalens,^55^ holograms,^56^ spectral filters^57^ and vortex beam generators.^58^ The full extent of metasurface capability cannot yet be utilized because heterogeneous metasurfaces are difficult to design as they are electrically large in the transverse plane and the number of free parameters can exceed 10^9^.^59^ Metasurface design currently is restricted to the either the design of periodic and quasi-periodic structures or to using the unit-cell approximation (where the inter-element coupling is approximated^60,61^).

The design of metasurfaces with DL is a problem that has received the most attention from researchers compared to other structures. The vast range of possible geometries, sensitivity to excitation conditions and the absence of established theoretical performance limits make this design problem challenging. Because of the involvement of a substrate and neighboring interactions, it is expected that the spectral response exhibits more diversity than that of individual nanoparticles. Additionally, a wider set of shapes can be considered as opposed to isolated nanoparticles. From the point of view of DL, these problems will thus need networks of larger representational capacity to reach acceptable accuracy and sophisticated inversion techniques.

**Fig. 5:**
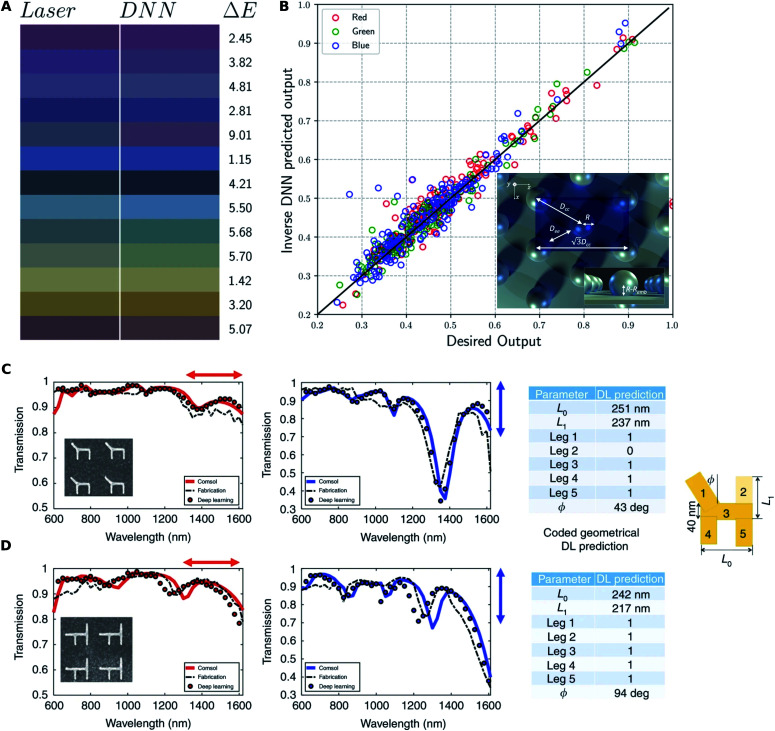
Experimental verification of DL-enabled design of periodic metasurfaces. (A) Comparison between the input test colours (left) and the colours produced by the structures fabricated using DNN predicted laser parameters (right). (B) Comparison between input RGB values (*x*-axis) and the values produced by the output laser parameters (*y*-axis) along with the ideal linear model (*y* = *x*). Reproduced with permission from ref. 62, under a Creative Commons Attribution 4.0 International license. (C and D) Two different gold nanostructures (shapes shown in the inset) are fabricated and their response to horizontal (red) and vertical (blue) polarization illumination is fed to the reverse DNN. Input to the reverse DNN (black dotted line) results in the predicted geometry tabulated on the right. The response predicted by the forward DNN (circles) and a full wave solver (solid lines) are shown. Reproduced with permission from ref. 22, under a Creative Commons Attribution 4.0 International license.

We can consider two types of periodic metasurfaces based on the periodicity: (1) subwavelength periodic metasurfaces, where the small periodicity ensures reflection and transmission in the zeroth order only; and, (2) metagratings, where multiple transmission and reflection orders exist. The most commonly encountered geometry encoding scheme is to encode the meta-atom (the unit-cell of a periodic metasurface) into a vector of parameters and the polarization-resolved transmittance and/or reflectance spectra are the response considered. DL based design is also indicated as most papers published on this type of structure report reasonable agreement between experimentally measured and numerically simulated responses. Fig. 5 shows the results reported by two papers where DL based inverse design has been experimentally validated.

An important subclass is the design of spectral filters (color filters) using such structures with the requirement of polarization-insensitivity. Baxter and co-workers^62^ reported an application of DL to the design of structural color resulting from a periodic nanoparticle array created by laser ablation. They used multiple interlinked DNNs (each trained separately), initialized inputs randomly and iterated to find a set of experimental parameters needed for a particular color. The performance of this technique is seen in Fig. 5A and B where the experimentally determined color of the inverse designed structures closely matches the targeted color. Some parameters may influence the observed color more than the others. Hemmatyar and co-workers^63^ experimentally demonstrated the use of hafnia (Hf0_2_) metasurfaces for vivid and high-purity colored pixels. The relative importance of each of the parameters of the structures was first determined through DL before finalizing the designs for experimental study. An autoencoder is used to obtain a dimensionality reduced representation of the spectra in the first step; a pseudoencoder network with a bottleneck layer then provides a quantitative estimate of the relative importance of each parameter. In their study, the authors found that the observed color is most sensitive to the periodicity parameter. The simulation work by Sajedian and co-workers^64^ and by Huang and co-workers^65^ used the technique of deep reinforcement learning. However, Sajedian and co-workers reported that the method takes a very long time to converge.

A generalization of the color filter design problem is to design structures for arbitrary spectral responses with polarization-sensitivity. The work by Malkiel and co-workers considered a chair shaped meta-atom as shown in Fig. 5C and D. The inversion is achieved by a reverse DNN that is directly trained, and a forward DNN is also trained for spectrum prediction. The verification shown in Fig. 5C and D is done by first fabricating an arbitrary structure and experimentally measuring its response. The experimental spectra are used as the input to the reverse DNN and the predicted inverse design is compared with the original design. The DL predicted structure is then used as the input to the forward DNN and an exact solver and these outputs are compared with the measured response. The close match in shapes and responses is seen and validates the DL based design approach.

Balin and co-workers^66^ applied DL to design and optimize a VO_2_ grating for smart window application. The grating was parametrized as a vector and a DNN was trained directly to predict the performance metrics of the smart window. This trained DNN was used to find a design by applying the classical trust region algorithm. The noteworthy feature of this work was the use of Bayesian training methods which result in clear uncertainty limits on the prediction of the forward DNN. The incorporation of prior information into the learning process using the Bayesian training ensured that overfitting did not occur even when the training dataset size was small. An alternative way to reduce the training dataset sizes involves dimensionality reduction (DR). Kiarashinejad and co-workers^67^ described a DR technique where a reduced representation of the input space is learned and useful information about the relative importance of parameters becomes evident. This technique was applied to the design of a reconfigurable optical metagrating enabling dual-band and triple-band optical absorption in the telecommunication window.

Ma and co-workers^19^ reported a DL-based technique for the design of chiral metamaterials where the meta-atom shape is parametrized as a one-dimensional vector. They reported a complex workflow which involves multiple networks with data flows designed to allow fully bidirectional operation (*i.e.* design parameters (or target spectra) can be input and spectra (or design parameters) can be output). Nadell and co-workers^68^ used a convolutional architecture for modeling a metasurface unit-cell and reported low validation errors. They also reported a fast inversion technique using only a forward DNN termed the fast forward dictionary search (FFDS).

A major limitation of the studies covered so far is the use of parameter vectors to encode shape. It requires the repetition of the train and test cycle for each new variant. Other ways to parametrize geometry exist. Inampudi and co-workers^20^ considered the larger set of fully closed shapes with polygon boundaries. Specifically, the shape of each unit is parameterized as a sixteen sided polygon with sixteen vertices whose positions vary in steps between some bounds. Each vertex can be represented as (*r*_*i*_, *θ*_*i*_), *i* = 1, …, 16 in polar coordinates. The polar angles *θ*_*i*_ of the vertices are uniformly distributed between 0 and 2π so that the shape of the unit is completely specified by the radius coordinates *r*_*i*_ alone. The chosen periodicity of the metagrating and the wavelength of incident light will result in a total of 13 propagating diffraction orders and the efficiency of diffraction into each of these orders is what the NN is trained for. The trained NN was finally used as a surrogate model in an optimization routine to demonstrate the inverse design capability.

The meta-atom shape can in fact be considered as an image with colors as indexing materials. This general form of a meta-atom was considered in the study by Sajedian and co-workers.^23^ They considered a convolutional neural network in association with a RNN. Their study reported only the forward NN development and needed a development time of several weeks. Although the final model is able to predict the response in a split-second, it remains unclear how well this trained model performs in an inverse design setting. Furthermore, we note that a large class of shapes are clearly impractical and thus the search has to be somehow constrained to the set of feasible geometries.

**Fig. 6:**
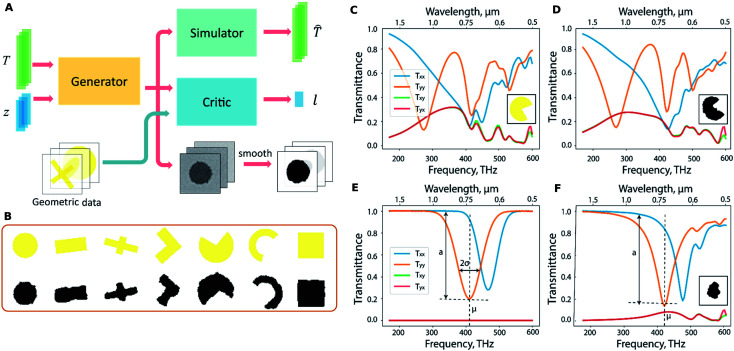
Generative NN based meta-atom design: (A) Architecture of the proposed network showing the sub-networks. (B) Test patterns (yellow) and the corresponding generated patterns (black) show the critic-network enabled guidance on structure generation. The spectra of a known geometry (depicted in the inset in yellow) seen in (C) are used to test the generative process which results in the structure shown in the inset of (D). The spectra show the successful inversion. For the desired spectrum shown in (E), the generative network yields the shape shown in the inset of (F). Reproduced with permission from ref. 25, ©2018, American Chemical Society.

The work by Liu and co-workers^25^ proposed the use of generative networks trained in an adversarial setting to perform inverse design without restricting the geometry to a smaller set. On the other hand, it uses a third network to ensure that the set does not grow too big. The architecture of the proposed method is seen in Fig. 6A and consists of three sub-networks. The simulator sub-network is the familiar forward NN. The generator accepts the spectra *T* and produces shapes depending on the spectra and a random noise vector *z*. Using the noise vector thus enables this network to learn a one-to-many mapping thus overcoming the problem of the tandem network. The generative process, however, must be somehow constrained to output feasible geometries which is accomplished with the critic sub-network. The critic sub-network is fed with a structure dictionary and is trained to recognize geometries similar to those in the dictionary. Fig. 6B shows a sample dictionary and its utility in nudging the generative process to adhere to feasible geometries. Fig. 6C–F show the ability of the trained generative network to find appropriate shapes given target spectra. The shapes chosen for this work were quite arbitrary (even including handwritten digit shapes). Jiang and co-workers^26^ reported an improved way to design the shape training dataset where realistic topologically complex shapes are used.

**Fig. 7:**
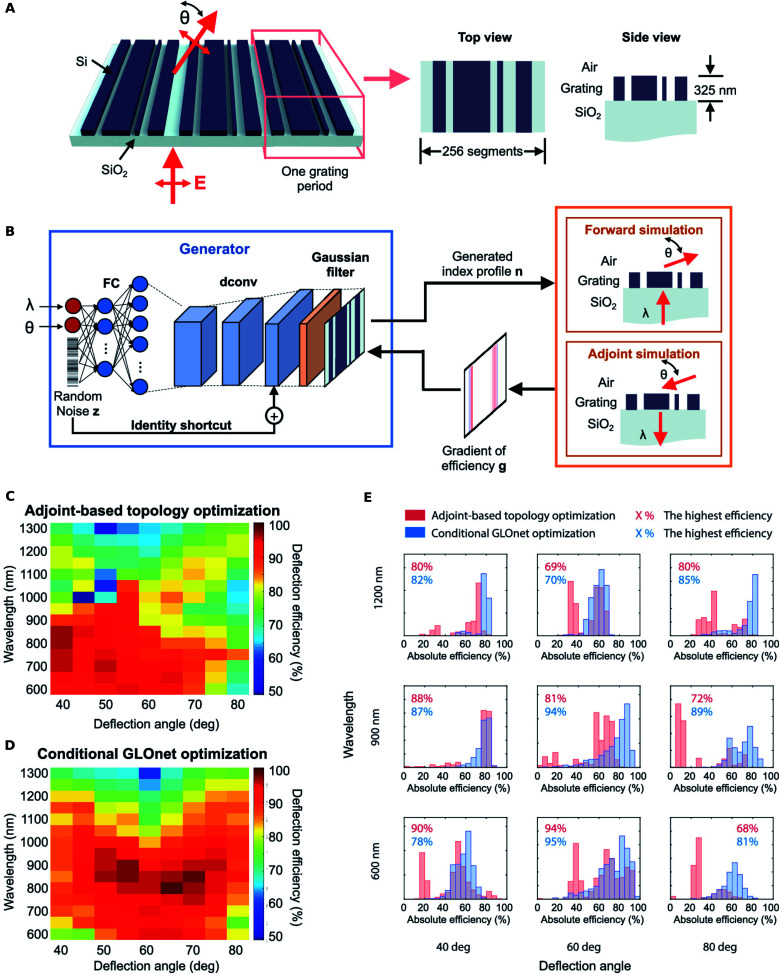
Global optimization based on a generative neural network (conditional GLOnet). (A) Schematic of the silicon metagrating being designed. (B) Schematic of the conditional GLOnet for metagrating generation and the loss construction. Performance comparison of adjoint-based topology optimization and conditional GLOnet optimization. (C and D) Plot of the best metagrating efficiency for devices operating with different wavelength and angle values designed using adjoint-based topology optimization and the conditional GLOnet respectively. (E) Efficiency histograms of devices designed using adjoint-based topology optimization (red) and conditional GLOnet optimization (blue). The highest device efficiencies in each histogram are also displayed. Reproduced with permission from ref. 34, ©2019, American Chemical Society.

The generation of the training dataset is often done by random sampling of the input space. In cases where this process is computationally costly, one is forced to resort to a smaller set which may unintentionally bias the trained NN. Jiang and co-workers^34,69^ reported a generative neural network based method which they titled as the “conditional GLOnet” (see Fig. 7A and B for the nanostructure schematic, the NN architecture and the hand-crafted loss) which delivers a group of globally optimal metagratings directly without the need for separate dataset generation, forward NN training and inversion steps. Beginning with a uniform sample across the input space, the algorithm iteratively converges towards a fruitful region of the design space. The algorithm can be considered as a search in the space of mappings, or equivalently, as the training of a generative network to output optimal devices for any random input. The training procedure involves a hand-crafted loss function that involves forward and adjoint electromagnetic simulations at each step. With metagratings operating across a range of wavelengths and angles as a model system, the authors' method outperformed adjoint-based topology optimization both in terms of quality of optima and runtime.

The authors generated 500 devices for each wavelength and reported the efficiencies of the best devices for the same wavelengths and deflection angles comparing their proposed method with a topology optimization method (see Fig. 7C and D). It is seen that, statistically, the best devices from the conditional GLOnet compare well with or are better than the best devices from adjoint-based optimization in most regimes; however, it did not optimally perform in certain regimes. The efficiency histograms from adjoint-based topology optimization and the conditional GLOnet for select wavelength and angle pairs show, in Fig. 7E, that the variance of the proposed method is better.

The training of generative networks is known to be problematic in the DL literature, specifically, training can get into endless loops with no subsequent improvement in performance. In two subsequent contributions by Liu and co-workers,^27,28^ the idea of generative networks was combined with Dimensionality Reduction (DR)^32,67,70^ which obviates the difficulties associated with adversarial generative training. Using a variational autoencoder, a latent space representation of the set of feasible geometries was developed. This latent space was then searched more efficiently using an evolutionary optimization method. Liu and co-workers^27^ reported the rapid design of a variety of metadevices for multiple functionalities using this method.

### 3.4 Integrated waveguides and passive components

Nanostructures and metadevices are beginning to play an important role in integrated photonics^71^ besides the fact that silicon photonic devices^72^ typically also contain features with sub-micron dimensions.^73,74^ The use of nanoscale features in silicon photonics introduces a vulnerability to fabrication related variations and defects which need to be well quantified. Several recent reports in the literature have focused on the application of DL to design problems in integrated photonics.

The application of dimensionality reduction to the design of integrated photonics devices achieves a functionality beyond that obtained through optimization runs. In a set of papers, Melati and co-workers proposed machine learning (ML) methodology that uses dimensionality reduction to create a map and to characterize a multi-parameter design space.^75^ Once created, this map can assist in several design and optimization tasks incurring a fraction of the computation cost of traditional optimization methods.

Hammond and co-workers^73^ proposed a new parameter extraction method using DL and demonstrated its applicability in extracting the true physical parameters of a fabricated Chirped Bragg Grating (CBG). Gostimirovic and co-workers^76^ reported the use of DL in the accelerated design of polarization-insensitive subwavelength grating (SWG) couplers on a SOI (silicon-on-insulator) platform. The model could optimize SWG-based grating couplers for either a single fundamental-order, polarization, or both. The surrogate model of the SWG reported by the authors worked 1830 times faster than exact numerical simulations with 93.2% accuracy of the simulations. Bor and co-workers^77^ introduced a new approach based on the attractor selection algorithm to design photonic integrated devices showing improved performance compared to traditional design techniques; specifically, an optical coupler and an asymmetric light transmitter were designed. Gabr and co-workers^78^ considered the design of four common passive integrated devices (waveguides, bends, power splitters and couplers) with a forward DNN; they reported split-second evaluation speeds with errors less than 2%.

Asano and co-workers^79^ reported an approach to optimizing the *Q* factors of two-dimensional photonic crystal (2D-PC) nanocavities based on deep learning. The training dataset consisted of 1000 nanocavities generated by randomly displacing the positions of many air holes in a base nanocavity and their *Q* values determined by an exact method. A trained DNN was able to estimate the *Q* factors from the air hole displacements with an error of 13% in standard deviation. The gradient of *Q* with respect to the air-hole displacement obtained by the trained NN enabled the design of a nanocavity structure with an extremely high *Q* factor of 1.58 × 10^9^. The authors claimed that the optimized design has a *Q* factor more than one order of magnitude higher than that of the base cavity and more than twice that of the highest *Q* factors ever reported so far for cavities with similar modal volumes. These results are a promising approach for designing 2D photonic crystal based integrated photonic devices. Zhang and co-workers^80^ reported a novel DL based approach to achieve spectrum prediction, parameter fitting, inverse design, and performance optimization for the design of plasmonic waveguide-coupled with cavity structure.

## 4 Perspectives on challenges and emerging developments

In the previous sections, we discussed the successful application of DL in the design of many kinds of photonic nanostructures and noted its potential to accelerate conventional design workflows and to enable unconventional workflows. Many problem domains have seen the application of DL for periods longer than the computational nanophotonics community. Examining the evolution of DL techniques in these other domains (primarily computer vision), the literature in the broader field of SciML,^3^ and multiple nanophotonics-specific preprints provides a perspective on current limitations and fruitful research directions. Broadly speaking, we can classify the challenges into two categories: (1) limitations germane to DL, like the inability to train from small datasets; and (2) limitations arising from applying DL to computational nanophotonics.

Although deep learning has enjoyed remarkable success, its success is empirical; a deep theoretical understanding of how it works and why it is successful remains elusive. The algorithms and networks of today are very complicated containing a very large number of parameters and strong nonlinear behavior, and it is thus not possible to determine exactly how the inputs lead to observed outputs (the “blackbox” problem). As a result, the following questions which naturally arise during the entire process do not have clear answers and require tedious trail and error:

1. What is the best choice of model architecture and how expressive should the model be?

2. What is the dataset size needed, how does this relate to generalization capability of the chosen network? How do we efficiently sample the domain?

3. How do we efficiently train the model, can we use physically meaningful losses and objectives?

4. How do we test the generalization ability of a trained DNN?

5. What exactly has the model learned from the data?

6. What steps should be taken to improve the model performance?

Although DL has become a very popular technique, it is safe to say that many computational photonics researchers will not be familiar with the intricate details and may not keep updated with the very rapid pace with which this field is progressing. Thus the burden of model development (including inversion schemes) is one of the major challenges. We focus on three key directions that will lead to reduction of the model development burden when used in isolation or in combination.

### 4.1 Dimensionality reduction

One way to reduce the model development burden is to develop a highly general model (*e.g.* a forward DNN which can predict the response of a wide class of shapes). Dimensionality reduction (DR) is a statistics/machine learning term that refers to the process of reducing the number of random variables under consideration by replacing the original set of numbers with a reduced set. Deep neural networks can achieve a nonlinear DR which can provide many advantages: (1) euclidian distance in the reduced space is a good measure of “similiarity” as we intuitively perceive it; (2) it is easier to perform searches in the reduced space. DR techniques can be applied to the structure space as well as the response space^67,70^ and to both spaces at the same time as well. DR is usually performed using a specially shaped DNN called the autoencoder (AE)^2^ which is characterized by the presence of a bottleneck layer. A popular variant of the deep autoencoder, the variational autoencoder (VAE),^81^ offers several advantages over the standard AE. The training of a VAE requires only a synthetic dataset of shapes or spectra and can be accomplished without the need for expensive EM simulations. A trained VAE can be split into an encoder and decoder and can be subsequently used as a generative network.

**Fig. 8:**
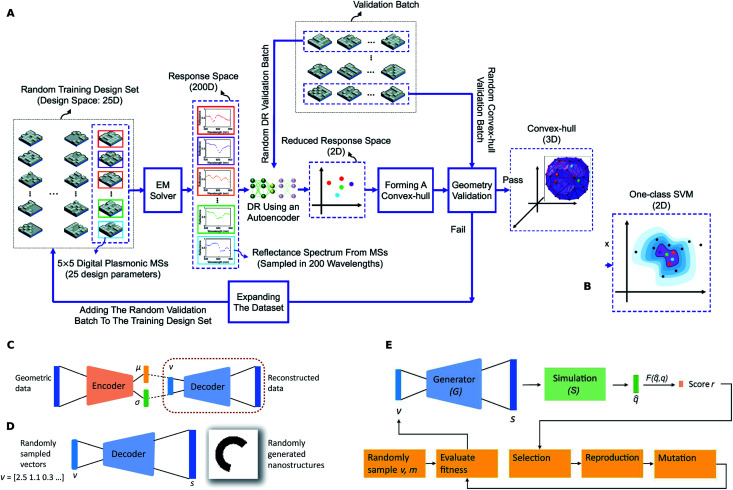
Dimensionality reduction in structure and response spaces. (A) Training algorithm for finding the convex-hull of the patterns in the latent response space. (B) A one-class SVM based non-convex boundary as an alternative to the convex hull. The actual dimensionality of the response space is not 2D as shown in the schematic. Reproduced with permission from ref. 32 under the Creative Commons license. (C) Training process for generating a latent representation of the structure (*i.e.* latent structure space) using a variational autoencoder (VAE). (D) After the training, the decoder encircled in (C) can be split-off and can act as a generator of geometric data given the latent vector input. (E) Flowchart of the VAE-ES framework where the evolutionary search occurs in the learned latent structure space. Reproduced with permission from ref. 28 under the Creative Commons license.

A DR representation of the spectral response of a class of geometries can be used to determine the range of responses possible from that class. Kiarashinejad and co-workers^32^ considered a checkerboard shaped geometry seen in Fig. 8A (each “pixel” can be “off” or “on”) and considered the set of all possible spectra. In the learned latent space of the spectral responses, they showed that a convex hull (a convex shaped boundary) can be determined without exhaustively calculating every spectrum. A tighter boundary using one-class support vector machine (Fig. 8B) can also be obtained similarly. Using this boundary shape allowed the authors to test whether a target response was achievable with the geometric class (the degree of feasibility can also be quantified).

A big limitation of DL based optimizations is that the structure shape is fixed beforehand and its parameters are adjusted. For every new shape, the entire process including dataset generation, model training, and hyper-parameter tuning has to be repeated. It is not known beforehand whether a given shape will be able to meet the target response. Liu and co-workers reported a DR technique to simultaneously search over a multiple number of shapes.^27,28^Fig. 8C shows the training of a VAE with a shape dataset where the encoder and decoder denote separate DNNs. The encoder network outputs a mean *μ* and a standard deviation *σ* vector from which we can sample a latent vector *v*. The decoder can be split-off after the training to serve as a generator of shapes given latent vector inputs (Fig. 8D). The specialty of the VAE is that for any given latent vector *v*, the generator will now output a “reasonable” looking shape that is a smooth hybrid between the shapes in the initial training dataset. An evolutionary search was then performed on the learned latent space using the flowchart seen in Fig. 8E. In an alternative paper, Liu and co-workers^27^ used a Compositional Pattern Producing Network (CPPN) as the shape generator. The CPPN produces higher quality shapes in comparison to a VAE decoder.

### 4.2 Acceleration of forward solvers

A common element in all DL methods is the requirement for dataset generation. Dataset generation requires the use of a forward solver which solves Maxwell's equations (or their simplified forms) repeatedly. Reduction in the computational cost of dataset generation will also significantly alleviate the model development burden. In recent years, a significant amount of effort has been directed towards the use of DL to accelerate partial differential equation (PDE) solvers.^3,83^ A particular attraction is that DL based PDE solvers may also be able to solve inverse problems without the need for extra effort.^83^

Trivedi and co-workers^84^ reported the acceleration of the finite difference frequency domain (FDFD) simulation of Maxwell's equations using data-driven models. An iterative solver such as the Generalized Minimal Residual (GMRES) algorithm is at the heart of FDFD solvers where a large sparse system of linear equations needs to be solved. The authors interfaced a DNN with a regular GMRES (that they call the data-driven GMRES). The data-driven GMRES preserved the same accuracy of a typical GMRES. The authors report an order of magnitude reduction in the number of iterations needed to reach convergence for the case of grating design.

**Fig. 9:**
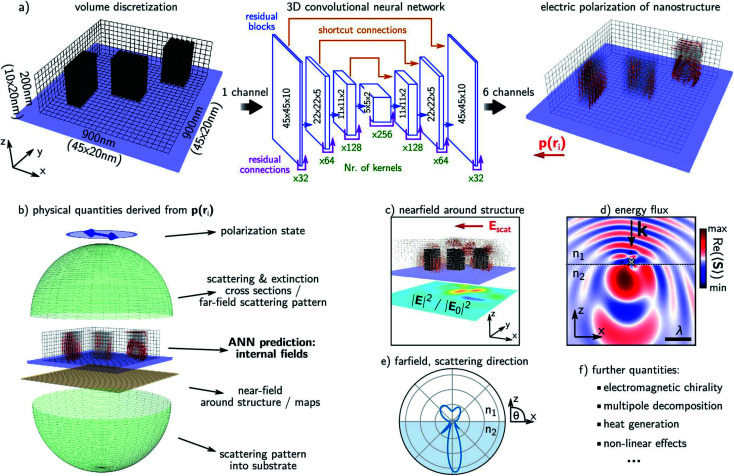
A forward DNN to predict the polarization density at every point in an arbitrary geometry. (a) The architecture of the DNN, the input and outputs and the volume discretization scheme of the 3D geometry. The principal layout of these blocks, the number of kernels and the layer dimensions are shown. (b–f) Various derived physical quantities that can be obtained from the output of the trained DNN are described. A glass substrate is assumed; illuminating light is assumed to be linearly polarized and monochromatic at 700 nm. Reproduced with permission from ref. 82, ©2020, American Chemical Society.

Wiecha and co-workers^82^ reported that DL can learn to predict the electromagnetic field quantities in an arbitrary geometry. Their report considers two-material systems with arbitrary placement of high-index inclusions in a vacuum matrix. As seen in Fig. 9a, the network architecture has a voxel-discretized rectangular region on which the input and the output are defined. The input specifies the inclusion of the high-index material and the output is a 6-dimensional vector at every voxel containing the *x*, *y* and *z* components of the complex (time-harmonic) electric field. Using the coupled dipole approximation, this can be converted into an electric polarization density *ρ*(*r*_*i*_) at the voxel. Various derived quantities can then be obtained using the CDA formalism as depicted in Fig. 9b–f. The main limitation of this demonstration is that the entire procedure has to be repeated for a different excitation frequency. Also, it is noted that the predictions are mostly qualitatively correct with a non-negligible probability of a very large error.

### 4.3 Transfer learning

An alternative way to reduce the burden of dataset generation and training is to capitalize on an already trained DNN. Transfer learning refers to the accelerated training of a DNN model on a smaller dataset using a parent DNN which has been trained on a similar (but not identical) learning task. All optical nanostructure design problems ultimately rely on the same set of well defined equations and thus in principle it should be possible to achieve transfer learning across design problems.

Qu and co-workers^85^ reported a study investigating the possibility of transfer learning in optics design problems. The first scenario examined was that of knowledge migration between very similar situations (in the authors' case, it was between the optical response of multilayered thin-films with different numbers of layers). The relative error rate was reduced by 50.5% (23.7%) when the source data come from 10-layer (8-layer) films and the target data come from 8-layer (10-layer) films. Secondly, the authors considered knowledge migration between seemingly different scenarios: between the optical response of multilayered spherical nanoparticles and multilayered thin-films where the relative error rate decreased by 19.7%. A third task involved learning multiple tasks simultaneously (predicting the optical response of multilayered thin-films with various total numbers of layers) where only a small training set was available for each task. The authors report that this strategy was only partially successful. The authors claim that their transfer learning framework was able to discover the aspects of underlying physical similiarity between problems.

## 5 Conclusions

The topical review has comprehensively surveyed the existing reports of deep learning based design of photonic nanostructures, the current limitations and some methods that are extending the reach of this technique. In this section, we look on some unaddressed problems in nanophotonics inverse design and in DL design methodology.

A wide variety of materials can be used in nanophotonics structures. The possible design space consisting of material/structure combinations is vast. A unified framework to explore the combined design space has not yet been reported. In the case of grating and metadevice design, only single material designs have been reported. Shape has a very strong influence on the optical properties of nanostructures. When shape is considered, the parameter space is quite vast including fractal^86–88^ and irregular shapes. More work is required in creating useful shape datasets with shapes that are topologically rich,^26^ yet experimentally realizable.^14^ Strongly coupled nanoparticle systems exhibit interesting spectral features^89^ and are invaluable in sensing applications.^90^ Collective behavior of multiple nanoparticles^91^ is a computationally challenging problem due to the increase in the number of free parameters. Fabrication constrained design^14^ and uncertainty quantification^92^ are extremely useful in the experimental realization of design nanostructures. DL techniques could prove invaluable in bridging the simulation–experiment gap and help avoid multiple iterations.

The landscape of deep learning in general, and SciML in particular, is fast evolving and techniques relevant to solving scientific problems are currently the subject of intense research.^3^ While early papers have relied on standard architectures and algorithms, it is anticipated that domain-specific architectures^3^ and algorithms would need to evolve to address harder problems (*e.g.* 3D geometries).

Writing for Nature, Riley^18^ points out the risks of using DL without proper checks and balances. In the field of optical nanostructures, fullwave simulations and experimental verification serve as ultimate checks, but it is entirely possible that researchers' efforts may get wasted if they are unaware of pitfalls of DL. It is conceivable that domain-specific architectures (where human knowledge can constrain DL) and efficient training routines may need to evolve to address intractable problems.

Sharing of domain-specific datasets between researchers is another avenue which will be very beneficial. Publicly available standard datasets (like the MNIST handwritten digits dataset) are invaluable when comparing the efficacy of various DL methodological alternatives. Only a select few papers reviewed here have links to code repositories and, in some cases, datasets. The ultimate success of a proposed methodology will depend on whether it enables the discovery of a design that can be physically realized. Nevertheless, performance improvement on standard datasets can be invaluable in guiding the methodology development. While innovative ideas have been proposed for inversion, it is not entirely clear whether reverse DNNs can discover better designs than conventional optimization methods;^30,31^ comparative studies on standard datasets will be invaluable in properly comparing different methodologies. Isolated nanoparticle design and multilayer thin-film designs can be such standard problems.

## Conflicts of interest

There are no conflicts to declare.

## Supplementary Material

## References

[cit1] LeCun Y., Bengio Y., Hinton G. (2015). Nature.

[cit2] Goodfellow I. , Bengio Y., Courville A. and Bach F., Deep Learning, MIT Press, Cambridge, Massachusetts, 2017

[cit3] Baker N. , Alexander F., Bremer T., Hagberg A., Kevrekidis Y., Najm H., Parashar M., Patra A., Sethian J., Wild S. and Willcox K., Workshop Report on Basic Research Needs for Scientific Machine Learning: Core Technologies for Artificial Intelligence, USDOE Office of Science (SC), Washington, D.C. (United States), technical report, 2019

[cit4] Massa A., Marcantonio D., Chen X., Li M., Salucci M. (2019). IEEE Antennas Wirel. Propag. Lett..

[cit5] Luna P. D., Wei J., Bengio Y., Aspuru-Guzik A., Sargent E. (2017). Nature.

[cit6] Curtarolo S., Hart G. L. W., Nardelli M. B., Mingo N., Sanvito S., Levy O. (2013). Nat. Mater..

[cit7] Winter R., Montanari F., Steffen A., Briem H., Noé F., Clevert D.-A. (2019). Chem. Sci..

[cit8] Zdeborová L. (2017). Nat. Phys..

[cit9] Le T. C., Winkler D. A. (2016). Chem. Rev..

[cit10] Chandrasekaran A., Kamal D., Batra R., Kim C., Chen L., Ramprasad R. (2019). npj Comput. Mater..

[cit11] Koenderink A. F., Alu A., Polman A. (2015). Science.

[cit12] Molesky S., Lin Z., Piggott A. Y., Jin W., Vucković J., Rodriguez A. W. (2018). Nat. Photonics.

[cit13] Campbell S. D., Sell D., Jenkins R. P., Whiting E. B., Fan J. A., Werner D. H. (2019). Opt. Mater. Express.

[cit14] Piggott A. Y., Petykiewicz J., Su L., Vučković J. (2017). Sci. Rep..

[cit15] Bellman R. , Dynamic Programming, Dover Publications Inc., Mineola, N.Y, 2003

[cit16] Yao K., Unni R., Zheng Y. (2019). Nanophotonics.

[cit17] Zhou J., Huang B., Yan Z., Bünzli J.-C. G. (2019). Light: Sci. Appl..

[cit18] Riley P. (2019). Nature.

[cit19] Ma W., Cheng F., Liu Y. (2018). ACS Nano.

[cit20] Inampudi S., Mosallaei H. (2018). Appl. Phys. Lett..

[cit21] Peurifoy J., Shen Y., Jing L., Yang Y., Cano-Renteria F., DeLacy B. G., Joannopoulos J. D., Tegmark M., Soljačić M. (2018). Sci. Adv..

[cit22] Malkiel I., Mrejen M., Nagler A., Arieli U., Wolf L., Suchowski H. (2018). Light: Sci. Appl..

[cit23] Sajedian I., Kim J., Rho J. (2019). Microsyst. Nanoeng..

[cit24] Zhang Q., Liu C., Wan X., Zhang L., Liu S., Yang Y., Cui T. J. (2019). Adv. Theory Simul..

[cit25] Liu Z., Zhu D., Rodrigues S. P., Lee K.-T., Cai W. (2018). Nano Lett..

[cit26] Jiang J., Sell D., Hoyer S., Hickey J., Yang J., Fan J. A. (2019). ACS Nano.

[cit27] Liu Z., Zhu D., Lee K.-T., Kim A. S., Raju L., Cai W. (2019). Adv. Mater..

[cit28] Liu Z. , Raju L., Zhu D. and Cai W., 2019, 18, arXiv:1902.02293 [physics]

[cit29] Hegde R. S. , Proceedings of SPIE 11105, Novel Optical Systems, Methods, and Applications XXII, San Diego, CA, USA, 2019

[cit30] Hegde R. S. (2019). Opt. Eng..

[cit31] Hegde R. S. (2020). IEEE J. Sel. Top. Quantum Electron..

[cit32] Kiarashinejad Y., Zandehshahvar M., Abdollahramezani S., Hemmatyar O., Pourabolghasem R., Adibi A. (2019). Advanced Intelligent Systems.

[cit33] Liu D., Tan Y., Khoram E., Yu Z. (2018). ACS Photonics.

[cit34] Jiang J. and Fan J. A., 2019, arXiv:1906.07843 [physics]

[cit35] Hu B., Wu B., Tan D., Xu J., Chen Y. (2019). Opt. Express.

[cit36] So S., Mun J., Rho J. (2019). ACS Appl. Mater. Interfaces.

[cit37] Maier S. A. , Plasmonics: Fundamentals and applications, 2007, pp. 1–223

[cit38] Barnes W. L., Dereux A., Ebbesen T. W. (2003). Nature.

[cit39] Permyakov D., Sinev I., Markovich D., Ginzburg P., Samusev A., Belov P., Valuckas V., Kuznetsov A. I., Luk B. S., Miroshnichenko A. E., Neshev D. N., Kivshar Y. S. (2015). Appl. Phys. Lett..

[cit40] He J., He C., Zheng C., Wang Q., Ye J. (2019). Nanoscale.

[cit41] Schallenberg U. B. (2006). Appl. Energy.

[cit42] Tikhonravov A. V., Dobrowolski J. A. (1993). Appl. Opt..

[cit43] Dobrowolski J. A., Tikhonravov A. V., Trubetskov M. K., Sullivan B. T., Verly P. G. (1996). Appl. Opt..

[cit44] Tikhonravov A. V. (1993). Appl. Opt..

[cit45] Anzengruber S. W., Klann E., Ramlau R., Tonova D. (2012). Appl. Opt..

[cit46] Zhao Y., Chen F., Shen Q., Zhang L. (2014). Prog. Electromagn. Res..

[cit47] Yang J.-m., Kao C.-y. (2001). Appl. Opt..

[cit48] Ebrahimi M., Ghasemi M. (2018). Opt. Quantum Electron..

[cit49] Janicki V., Sancho-parramon J., Zorc H. (2008). Thin Solid Films.

[cit50] Becker H., Tonova D., Sundermann M., Ehlers H., Günster S., Ristau D. (2014). Appl. Opt..

[cit51] Chen Y., Zhu J., Xie Y., Feng N., Liu Q. H. (2019). Nanoscale.

[cit52] Storn R., Price K. (1997). J. Global Optim..

[cit53] Genevet P., Capasso F. (2015). Rep. Prog. Phys..

[cit54] Ding F., Pors A., Bozhevolnyi S. I. (2018). Rep. Prog. Phys..

[cit55] Khorasaninejad M., Chen W. T., Devlin R. C., Oh J., Zhu A. Y., Capasso F. (2016). Science.

[cit56] Zheng G., Mühlenbernd H., Kenney M., Li G., Zentgraf T., Zhang S. (2015). Nat. Nanotechnol..

[cit57] Vashistha V., Vaidya G., Hegde R. S., Serebryannikov A. E., Bonod N., Krawczyk M. (2017). ACS Photonics.

[cit58] Tang S., Cai T., Wang G.-m., Liang J.-g., Li X., Yu J. (2018). Sci. Rep..

[cit59] Byrnes S. J., Lenef A., Aieta F., Capasso F. (2015). Opt. Express.

[cit60] Donda K. D., Hegde R. S. (2017). Prog. Electromagn. Res..

[cit61] Donda K. D., Hegde R. S. (2019). Prog. Electromagn. Res..

[cit62] Baxter J., Calà Lesina A., Guay J.-M., Weck A., Berini P., Ramunno L. (2019). Sci. Rep..

[cit63] Hemmatyar O., Abdollahramezani S., Kiarashinejad Y., Zandehshahvar M., Adibi A. (2019). Nanoscale.

[cit64] Sajedian I., Badloe T., Rho J. (2019). Opt. Express.

[cit65] Huang Z., Liu X., Zang J. (2019). Nanoscale.

[cit66] Balin I., Garmider V., Long Y., Abdulhalim I. (2019). Opt. Express.

[cit67] Kiarashinejad Y., Abdollahramezani S., Zandehshahvar M., Hemmatyar O., Adibi A. (2019). Adv. Theory Simul..

[cit68] Nadell C. C., Huang B., Malof J. M., Padilla W. J. (2019). Opt. Express.

[cit69] Jiang J., Fan J. A. (2019). Nano Lett..

[cit70] Kiarashinejad Y. , Abdollahramezani S. and Adibi A., 2019, arXiv:1902.03865 [physics, stat]

[cit71] Cheben P., Halir R., Schmid J. H., Atwater H. A., Smith D. R. (2018). Nature.

[cit72] Michaels A., Wu M. C., Yablonovitch E. (2020). IEEE J. Sel. Top. Quantum Electron..

[cit73] Hammond A. M., Potokar E., Camacho R. M. (2019). OSA Continuum.

[cit74] Hammond A. M., Camacho R. M. (2019). Opt. Express.

[cit75] Melati D., Grinberg Y., Kamandar Dezfouli M., Janz S., Cheben P., Schmid J. H., Sánchez-Postigo A., Xu D.-X. (2019). Nat. Commun..

[cit76] Gostimirovic D., Ye W. N. (2019). IEEE J. Sel. Top. Quantum Electron..

[cit77] Bor E., Alparslan O., Turduev M., Hanay Y. S., Kurt H., Arakawa S., Murata M. (2018). Opt. Express.

[cit78] Gabr A. M., Featherston C., Zhang C., Bonfil C., Zhang Q.-J., Smy T. J. (2019). J. Opt. Soc. Am. B.

[cit79] Asano T., Noda S. (2018). Opt. Express.

[cit80] Zhang T., Wang J., Liu Q., Zhou J., Dai J., Han X., Zhou Y., Xu K. (2019). Photonics Res..

[cit81] Kingma D. P. and Welling M., 2013, arXiv:1312.6114 [cs, stat]

[cit82] Wiecha P. R., Muskens O. L. (2020). Nano Lett..

[cit83] Lu L. , Meng X., Mao Z. and Karniadakis G. E., 2019, arXiv:1907.04502 [physics, stat]

[cit84] Trivedi R., Su L., Lu J., Schubert M. F., Vuckovic J. (2019). Sci. Rep..

[cit85] Qu Y., Jing L., Shen Y., Qiu M., Soljačić M. (2019). ACS Photonics.

[cit86] Gottheim S., Zhang H., Govorov A. O., Halas N. J. (2015). ACS Nano.

[cit87] Tang S., He Q., Xiao S., Huang X., Zhou L. (2015). Nanotechnol. Rev..

[cit88] Hegde R. S., Khoo E. H. (2016). Plasmonics.

[cit89] Luk’yanchuk B., Zheludev N. I., Maier S. A., Halas N. J., Nordlander P., Giessen H., Chong C. T. (2010). Nat. Mater..

[cit90] Mesch M., Weiss T., Schäferling M., Hentschel M., Hegde R. S., Giessen H. (2018). ACS Sens..

[cit91] Auguié B., Barnes W. L. (2008). Phys. Rev. Lett..

[cit92] Tripathy R. K., Bilionis I. (2018). J. Comput. Phys..

